# Cellular and Molecular Mechanisms Underlying Pain Chronicity

**DOI:** 10.3390/cells12081126

**Published:** 2023-04-11

**Authors:** Manuela Simonetti, Daniela Mauceri

**Affiliations:** 1 Institute of Pharmacology, Medical Faculty Heidelberg, Heidelberg University, Im Neuenheimer Feld 366, 69120 Heidelberg, Germany; 2 Department of Neurobiology, Interdisciplinary Centre for Neurosciences (IZN), Heidelberg University, Im Neuenheimer Feld 366, 69120 Heidelberg, Germany; 3 Department Molecular and Cellular Neuroscience, Institute of Anatomy and Cell Biology, University of Marburg, Robert-Koch-Str. 8, 35032 Marburg, Germany

**Keywords:** molecular mechanisms, chronic pain, genetic, epigenetic, post-translational regulation, nociceptors, pain circuits

Chronic pain affects a significant amount of the population and is responsible for vast worldwide socio-economic costs [1]. Moreover, conventional therapies are not very effective in reducing pain [2]; in fact, clinicians report only 50 percent pain relief among patients who respond to treatments. Since chronic pain is not a pure prolongation of acute pain, but is based on structural and functional alterations of neural circuity [3], understanding the molecular mechanisms underlying these alterations is crucial for developing new effective therapies. Although progress has been made in describing the mechanisms underlying pain, many essential questions on the molecular and cellular players driving chronic pain still need to be answered. Thus, chronic pain continues to pose a challenge to preclinical and clinical researchers. This Special Issue, featuring seven original research articles and four reviews, discusses multiple pain models and offers insights at different levels: from molecular to cellular, from transcriptional to post-translational, from synaptic to circuitry. Further, diverse experimental tools, which will positively impact pain research, are offered to the reader. The studies presented here not only push forward pain research, but they also expand the current understanding of different pain modalities.

Chronic pain can have different aetiologies. This Special Issue discusses, for example, chronic pain due to nerve injury [4,5], chronic pain associated with a genetic disease [6], or chronic pain associated with cancer [7]. Hirth et al. characterized two mouse models (KPC and KPPC models) based on the most common genetic alteration found in human pancreatic cancer tissues (i.e., p53 and Kras) and suitable to study pain associated with pancreatic ductal adenocarcinoma (PDAC), the most prevalent type of pancreatic cancer [8,9]. They found that these models resemble characteristic of human pancreatic cancer, such as duct-like structure, almost absence of necrosis, neuronal remodeling (i.e., hypertrophy and increased nerve fibres density), marked neuroinflammation, as well as an exponential increase in cancer-associated pain with the disease progression in the KPC model [7]. Interestingly, the authors found the upregulation of several cytokines whose role in mediating the interaction between cancer and nerve fibres or microenvironment is still unknown. These could represent new mediators involved in the progression of both cancer and cancer–associated pain [7]. 

An important pitfall for the development of more efficient therapies is the difficulty in translating key findings derived from animal models to humans. In this view, the access and use of human pluripotent stem cells have surely enriched the pain field and will continue to provide important translational insights. The original article by Schrenk-Siemens and co-authors describes the generation of human stem cells-derived nociceptor-like cells [10]. By varying the differentiation protocol, the authors produced different nociceptive subpopulations with different nociceptive properties. Among the diverse described protocols, one, in particular, generated a highly homogenous population of nociceptive sensory neurons [10]. Thus, this article provides a novel, powerful translational tool to mechanistic studies of sensitization processes [10]. 

Neurostimulation approaches, such as transcranial direct current stimulation (tDCS), are emerging as new therapeutic approaches to treat refractory forms of neuropathic pain in rodent models, as well as in clinical studies [11,12]. Whereas most of the previous studies have focused on motor cortex stimulation, Li et al. showed that, also, the repetitive neurostimulation of the posterior insula (PI tDCS) attenuates the development of nerve injury-induced neuropathic allodynia and reverses the chronically established allodynia for weeks, which mainly employs the descending opioid system [4]. Indeed, they found that PI tDCS induced suppression of activity in several pain-associated brain regions, as well as the spinal cord, detected as a reduction in c-Fos-positive neurons in these areas [4].

Patients affected by neuropathic or inflammatory chronic pain are burdened by hypersensitivity to mechanical stimuli. Among many possible contributing factors, spinal disinhibition seems to play a prominent role. The original research article by Liu and co-authors specifically studied the contribution of presynaptic GABAergic inhibition in inflammatory mechanical hypersensitivity, taking advantage of a mouse transgenic model carrying a conditional deletion of GABAA in NaV1.8-positive sensory neurons [13]. With a combination of behavioral, molecular, and histological techniques, the authors described how mice lacking presynaptic inhibition developed reduced allodynia in response to punctuated, but not dynamic, stimuli [13]. Further, they correlated their findings to the number of cells activated in the dorsal horn assessed via the presence of c-fos mRNA or c-Fos proteins [13]. Of note, for their analysis, the authors additionally developed an ad hoc system for image analyses, which may be beneficial for many experimental researchers to standardize quantifications, and Liu et al. made these freely available [13]. 

Pain is a hallmark of Fabry disease (FD), a rare genetic disorder caused by a deficiency of the enzyme alpha-galactosidase A (GLA). Deficiency of GLA leads to the accumulation of globotriaosylceramide in dorsal root ganglia neurons. There are still vast gaps in understanding the mechanisms linking such accumulation to the pain experienced by FD patients. The work of Spitzel and co-authors characterized the cellular, molecular, and behavioral phenotype of a knockout GLA mouse line, which mimics FD [6]. They carried out their analyses by differentiating between young and old mice to further understand if and to which extent age may influence certain pathological aspects. Spitzel et al. indeed showed that, from a behavioral viewpoint, the GLA KO mice recapitulated what was observed in FD patients [6]. Further, they detected alterations in the expression levels of components of the molecular machinery responsible for inflammatory and immune responses [6]. In sum, the authors provide data supporting a mechanistic link between GLA dysfunction, altered immune response, and pain [6]. 

Modulation of inflammatory signaling was also found in a model of chronic pain induced by nerve injury [5]. Using a new conditional mouse line, which loses ß2-AR exclusively in microglial cells, Damo et al. demonstrated that specific activation of the microglial ß2 adrenergic receptor can modulate nerve injury-associated neuropathic pain by inducing a structural and functional change in microglial cells compared with an activated state associated to the nerve injury model used in the study (spared nerve injury, SNI) [5]. Interestingly, this alteration of microglia was associated, at least in vitro, with changes in cytokine release [5]. Application of a ß2 agonist to a culture of activated primary microglia induced a reduction in pro-inflammatory cytokines and an increase in the release of anti-inflammatory cytokines [5].

c-Fos is commonly used as a marker for active neurons in pain research and beyond [14,15,16]. Indeed, two articles in this Special Issue use this approach to identify active neurons [4,13]. Nevertheless, manually counting Fos-positive neurons is time-consuming and may largely be influenced by human bias, subjectivity, and variability. For these reasons, Beretta et al. developed an open source tool for ImageJ/Fiji, called Quanty-cFos, for unbiased counting of cells that are either Fos-positive or that express c-Fos mRNA [17]. Importantly, Quanty-cFos lacks human bias and allows reproducibility across different experiment counting cells in an automated or semi-automated way [17]. Furthermore, the study provides in-depth, step-by-step teaching videos for a quick and efficient application of the tool to other stainings, which are also performed also by non-experts [17]. 

In addition to the original research articles, this Special Issue contains four review articles, discussing different aspects of chronic pain. 

Mandel and Agarwal summarized and highlighted the involvement of a particular form of post-translational modification (PTMs), called SUMOylation, in the onset and progression of neurodegenerative diseases (NDDs) [18]. Post-translational modifications are essential to maintain neuronal homeostasis and for several cellular functions [19,20]. Proper synaptic and cognitive functions require the reversible and dynamic conjugation of a small ubiquitin-like modifier (SUMO) to specific substrates, and aberrant SUMOylation is frequently associated with NDDs, such as diabetic peripheral neuropathy (DPN), a chronic complication of diabetes. DPN can be characterized by neuropathic pain. Interestingly, different studies have identified, as SUMOylation targets, several ion channels involved in pain transmission, such as sodium channels 1.7 (Nav1.7), transient receptor potential V1 (TRPV1), and multiple voltage-gated potassium channels [18].

In addition to the original article by Damo et al. [5], the special role of glial cells in pain is also highlighted in the review of Damo and Simonetti, which discusses the current knowledge on the involvement of developmental molecules, such as Wnt, ephrins, and semaphorins, in the pathogenesis and progression of chronic pain, both from the neuronal, as well as glial, point of view [21]. Indeed, receptors and ligands of these pathways are expressed in a wide variety of neuronal and glial cells. Wnt, ephrin, and semaphorin signaling enhance neuronal excitability, peripheral sensitization, synaptic plasticity, as well as the production and release of inflammatory cytokines [21].

Sensitization and maladaptive plasticity of the cellular units in the pain circuitry is known to be sustained by changes in their transcriptional profile [22]. Epigenetic mechanisms play a pivotal role in the modulation of transcription in adaptive processes in the nervous system. Indeed, the last decade has seen the birth and fast growth of the pain epigenetic field of research, involving the characterization of several epigenetic mediators involved in pain and the identification of their downstream targets [23]. In this Special Issue, Mauceri provides, first, an overview of the major epigenetic processes and their molecular mediators—DNA methylation, histone post-translational modifications, and non-coding RNAs—followed by a discussion of the role of such mediators in different forms of chronic pain [24].

An important aspect of pain research focuses on the discovery or improvement of therapeutic approaches. This is a timely and critical issue, as therapies to prevent or handle chronic pain are still largely unsatisfactory. The review presented by Chen and co-authors focuses on neuropathic pain, arising after spinal cord injury (SCI), and it critically discusses the efficacy of activity-based interventions (ABI) in preclinical studies [25]. The review thoroughly compares the different ABI approaches and makes an effort to draw parallels and differences in regards to distinct parameters, such as duration or intensity [25]. As interventions based on physical activity are the main rehabilitative approach in the treatment of SCI patients, this review provides readers with the necessary knowledge to understand the scientific rationale for this therapeutic approach and opens avenues for future research and interventional directions [25].

The studies presented in this Special Issue highlight new aspects underlying the mechanisms of chronic pain of different origins, while also providing starting points for a better understanding of pain signaling and, finally, for the development of new therapies. At the same time, this Special Issue offers a glimpse into the complexity of chronic pain. For example, the dynamic molecular landscape involved in pain chronicity is reflected in two reviews, highlighting post-translational and epigenetic modifications of numerous proteins. Moreover, the interaction between the nervous system and immune mediators, namely, cytokines, is challenged in three papers, which deal with three different types of chronic pain, associated with nerve injury [5], cancer [7] and a genetic disease [6]. Furthermore, in their review, Damo and Simonetti emphasize, once again, the important role of cytokines as modulators of chronic pain [21]. This suggests that there are mechanisms common to the different forms of pain, and it lays the basis for subsequent studies aimed at identifying new common targets for the development of effective therapies. Finally, two studies in this Special Issue describe the development of new tools for image analysis, which is currently in high demand [13,17]. These systems, made freely available to the public, allow standardization of protein or mRNA quantification and avoid biased and time-consuming manual counting, while amplifying reproducibility among experiments through automatic cell counting.

In conclusion, the chronic pain field continues to pose stimulating research questions. This Special Issue examines several pain models and offers insights at various levels, providing the reader with valuable, timely updates in such a complex scenario.
